# Prediction of homologous recombination deficiency from Oncomine Comprehensive Assay Plus correlating with SOPHiA DDM HRD Solution

**DOI:** 10.1371/journal.pone.0298128

**Published:** 2024-03-25

**Authors:** Jun Kang, Kiyong Na, Haeyoun Kang, Uiju Cho, Sun Young Kwon, Sohyun Hwang, Ahwon Lee

**Affiliations:** 1 Department of Hospital Pathology, Seoul St. Mary’s Hospital, College of Medicine, Catholic University of Korea, Seoul, Korea; 2 Department of Pathology, Kyung Hee University College of Medicine, Kyung Hee University Hospital, Seoul, Republic of Korea; 3 Department of Pathology, CHA Bundang Medical Center, CHA University, Seongnam, Korea; 4 Department of Pathology, St. Vincent’s Hospital, College of Medicine, The Catholic University of Korea, Seoul, Korea; 5 Department of Pathology, Dongsan Hospital, School of Medicine, Keimyung University, Daegu, South Korea; 6 CHA Future Medicine Research Institute, CHA Bundang Medical Center, Seongnam, Korea; 7 Cancer Research Institute, Catholic University of Korea, Seoul, Korea; CNR, ITALY

## Abstract

**Objective:**

Poly(ADP-ribose) polymerase (PARP) inhibitors are used for targeted therapy for ovarian cancer with homologous recombination deficiency (HRD). In this study, we aimed to develop a homologous recombination deficiency prediction model to predict the genomic integrity (GI) index of the SOPHiA DDM HRD Solution from the Oncomine Comprehensive Assay (OCA) Plus. We also tried to a find cut-off value of the genomic instability metric (GIM) of the OCA Plus that correlates with the GI index of the SOPHiA DDM HRD Solution.

**Methods:**

We included 87 cases with high-grade ovarian serous carcinoma from five tertiary referral hospitals in Republic of Korea. We developed an HRD prediction model to predict the GI index of the SOPHiA DDM HRD Solution. As predictor variables in the model, we used the HRD score, which included percent loss of heterozygosity (%LOH), percent telomeric allelic imbalance (%TAI), percent large-scale state transitions (%LST), and the genomic instability metric (GIM). To build the model, we employed a penalized logistic regression technique.

**Results:**

The final model equation is -21.77 + 0.200 × GIM + 0.102 × %LOH + 0.037 × %TAI + 0.261 × %LST. To improve the performance of the prediction model, we added a borderline result category to the GI results. The accuracy of our HRD status prediction model was 0.958 for the test set. The accuracy of HRD status using GIM with a cut-off value of 16 was 0.911.

**Conclusion:**

The Oncomine Comprehensive Assay Plus provides a reliable biomarker for homologous recombination deficiency.

## Introduction

Homologous recombination repair (HRR) is a DNA repair mechanism that restores DNA double-strand breaks (DSBs) in cells. This mechanism is essential for maintaining genomic stability and preventing the accumulation of DNA damage that can lead to mutations and other genetic alterations. When HRR is impaired, such as through mutations in genes involved in this repair pathway, it can lead to a condition known as homologous recombination deficiency (HRD). HRD has been found to be associated with an increased risk of developing certain types of cancer, including ovarian, breast, and prostate cancer [1, 2]. The genes most commonly associated with HRD are *BRCA1* and *BRCA2*, which are tumor suppressor genes that play a critical role in HRR [3]. Mutations in these genes can impair the HRR pathway, leading to an increased risk of developing cancer. Other genes involved in HRR, such as *PALB2* and *RAD51*, have also been linked to HRD and an increased cancer risk [4, 5].

Poly(ADP-ribose) polymerase (PARP) inhibitors such as olaparib and talazoparib are a type of targeted therapy that work by inhibiting the function of PARP1, an enzyme that is involved in the repair of single-strand DNA breaks (SSBs). PARP inhibitors have shown clinical efficacy in BRCA1/2 mutant ovarian cancer, breast cancer, and prostate cancer [6–9]. These drugs have demonstrated promising results in clinical trials and have been approved by regulatory agencies for the treatment of certain types of cancer.

HRD can lead to abnormal DSB repair and result in genomic scars, which are large-scale genomic alterations that can be quantified by counting the number of occurrences. There are several types of genomic scars associated with HRD, including large-scale loss of heterozygosity (LOH) [10], telomere allelic imbalance (TAI) [11], and large-scale state transitions (LST) [12]. The HRD score is a quantification of these genomic scars and is used to identify patients who may benefit from treatment with PARP inhibitors [13, 14]. The HRD score is calculated based on the occurrence of these genomic scars. These tests, such as the Myriad MyChoice^®^ CDx and FoundationOne^®^ CDx tests, have been approved by regulatory agencies as companion diagnostics for PARP inhibitor treatment in patients with ovarian and prostate cancer [6, 8, 15, 16].

The SOPHiA DDM HRD Solution is an HRD test that identifies HRR mutations through targeted sequencing and measures genomic instability (GI) through a combination of low-pass whole-genome sequencing and a deep-learning algorithm [17]. The GI index is a measure of genomic stability of the SOPHiA DDM HRD Solution. This index is based on the analysis of the genome-wide patterns of copy number variations (CNVs) and is used to determine the level of GI in a tumor sample. A high GI index is associated with HRD tumors [17].

The Oncomine Comprehensive Assay (OCA) Plus is a targeted next-generation sequencing (NGS) assay designed to detect genetic alterations in solid tumors. The HRD score provided by the OCA Plus includes (1) percent LOH (%LOH), which estimates the fraction of the genome with LOH identified using genomic segmentation; (2) percent TAI (%TAI), which estimates the fraction of the genome with allelic imbalance or unequal contribution from the two alleles in the telomeres identified using genomic segmentation; and (3) percent LST (%LST), which estimates the fraction of the genome with unequal copy numbers in adjacent segments identified using genomic segmentation. These values range from 0 to 100. The genomic instability metric (GIM) is a proprietary measurement that quantifies genomic scarring associated with HRD [18].

As a centralized test, Myriad MyChoice^®^ CDx may pose challenges in areas where access to such centralized facilities is limited, making it less practical for routine patient diagnostics. Furthermore, the stringent DNA quality requirements associated with Myriad MyChoice^®^ CDx could introduce technical constraints, potentially limiting its applicability in settings where meeting these requirements poses difficulties. Conversely, the OCA Plus and SOPHiA DDM HRD Solution tests are in-house testing methodologies. Administering tests in-house allows for greater convenience within healthcare institutions where patients are receiving care, addressing the practical challenges associated with centralized testing and ensuring a more patient-centric testing environment [19].

In this study, we aimed to develop an HRD prediction model to predict the GI index of the SOPHiA DDM HRD Solution from the OCA Plus. We also tried to find a cut-off value for the GIM of the OCA Plus that correlates with the GI index of the SOPHiA DDM HRD Solution.

## Materials and methods

### Sample collection

We included 87 cases of high-grade ovarian serous carcinoma from five tertiary referral hospitals in the Republic of Korea. All cases had been tested with the OCA Plus NGS panel for clinical purpose at the hospitals where patients were treated. We excluded the cases that failed to analyze HRD scores provided by the OCA Plus. In all cases, we confirmed the clinical information and tissue diagnosis and we selected paraffin blocks for the SOPHiA DDM HRD Solution. We cut all formalin-fixed paraffin-embedded (FFPE) tissue samples to a thickness of 5 μm. We sent 10 sections to the institution in the Republic of Korea that performs the SOPHiA DDM HRD Solution. The data were collected from December 16, 2022, to February 7, 2023, and the information that could identify individual participants was not accessible during or after the data collection. The need for informed consent was waived by the Institutional Review Board of the CHA Bundang Medical Center, CHA University (2023-01-010-001) and the Catholic University of Seoul Saint Mary’s Hospital (KC18TNSI0361).

### Genomic DNA and RNA isolation and measurement

We extracted genomic nucleic acids by utilizing the RecoverAll^™^ Total Nucleic Acid Isolation Kit from Invitrogen^™^. We quantified and assessed the quality of DNA by using the NanoDrop^™^ 2000 spectrophotometer from Thermo Scientific and the Qubit^™^ fluorometer from Invitrogen^™^. We employed the Qubit^™^ fluorometer with the Qubit^™^ dsDNA HS test kit and the Qubit^™^ dsRNA HS test kit, following the manufacturer’s specified protocols.

### OCA Plus

We performed all manual library preparation by using the OCA Plus system (Thermo Fisher Scientific), following the manufacturer’s instructions. We conducted the multiplex polymerase chain reaction (PCR) amplification with an approximate DNA concentration of 20 ng. Prior to PCR amplification, we carried out the deamination reaction in the OCA Plus by using Uracil-DNA Glycosylase, heat labile (Thermo Fisher Scientific).

For sequencing, we loaded the prepared libraries onto Ion 550 Chips (Thermo Fisher Scientific) according to the manufacturer’s instructions and processed them using the Ion Chef System. We used the Ion S5 XL Sequencer (Thermo Fisher Scientific) for sequencing. We aligned the data to the human genome assembly 19, which served as the standard reference genome in the Ion Reporter Software (v. 5.18) (Thermo Fisher Scientific). Hospital B utilized the customized variability control informatics baseline (VCIB) for copy number analysis. The GIM was obtained from the Ion Reporter Software (v. 5.20).

### GI score prediction modeling

We developed an HRD prediction model that aimed to predict the GI index of the SOPHiA DDM HRD Solution. The training set consisted of cases from hospital A, while the test set comprised cases from the other hospitals. The predictor variable used in the model was the HRD score, which included %LOH, %TAI, %LST, and the GIM, provided by the OCA Plus. To build the model, we employed a penalized logistic regression technique. We selected the model through repeated fivefold cross-validation on a grid of hyperparameters: λ (10^−5^, 10^−4^, 10^−3^, 10^−2^, and 10^−1^) and α (0.0, 0.25, 0.5, 0.75, and 1.0).

### Assessing model performance

We estimated the performance of the prediction based on the area under the curve of the receiver operating characteristic curve (AUROC) for the GI status and the HRD status. We considered the GI status to be positive when the GI index exceeded 0. On the other hand, we considered the HRD status to be positive if there was a BRCA1/2 pathogenic variant or if the GI status was positive. We conducted the modeling and assessment of model performance using the tidymodels and glmnet R packages. A flowchart of the study is presented in S1 Fig.

In accordance with the journal’s guidelines, we will provide our data for independent analysis by a team selected by the Editorial Team for the purposes of additional data analysis or to reproduce this study in another center (if requested).

### Research ethics and patient consent

The study was approved by the Institutional Review Board of the CHA Bundang Medical Center, CHA University (2023-01-010-001), and the Catholic University of Seoul Saint Mary’s Hospital (KC18TNSI0361), where this study was organized.

## Results

### Patients

The average age of the patients was 59.3 years. The patients were distributed across International Federation of Gynecology and Obstetrics (FIGO) stages as follows: 8.1% in Stage 1, 7.0% in Stage 2, 73.3% in Stage 3, and 11.6% in Stage 4. Hospital A contributed the most cases (55, accounting for 63.2% of the total). There were no significant differences in patient age and the FIGO stage between the training set and the test set (Table 1).

**Table 1:** Cases summary.

	Overall	Training set	Test set	p value
**Number of patients**	87	55	32	
**Age (mean (SD))**	59.3 (10.3)	59.2 (10.5)	59.4 (10.2)	0.946
**FIGO stage (%)**				0.875
**1**	7 (8.1)	5 (9.3)	2 (6.2)	
**2**	6 (7.0)	3 (5.6)	3 (9.4)	
**3**	63 (73.3)	40 (74.1)	23 (71.9)	
**4**	10 (11.6)	6 (11.1)	4 (12.5)	
**Institution (%)**				
**Hospital A**	55 (63.2)	55 (100.0)	0 (0.0)	
**Hospital B**	15 (17.2)	0 (0.0)	15 (46.9)	
**Hospital C**	10 (11.5)	0 (0.0)	10 (31.2)	
**Hospital D**	4 (4.6)	0 (0.0)	4 (12.5)	
**Hospital E**	3 (3.4)	0 (0.0)	3 (9.4)	

SD, standard deviation; FIGO, The International Federation of Gynecology and Obstetrics

### SOPHiA DDM HRD Solution

The HRD status was positive in 56 cases (64.4%), negative in 23 cases (26.4%), and undetermined in 8 cases (9.2%). The GI status was positive in 50 cases (57.5%), negative in 27 cases (31.0%), and undetermined in 10 cases (11.5%). The BRCA status was positive in 28 cases (32.2%), negative in 45 cases (51.7%), and undetermined in 14 cases (16.1%).

### OCA Plus sequencing

The mean of the average base coverage was 2469.64. The mean of the median absolute pairwise difference (MAPD) was 0.24. The MAPD measures read coverage noise detected across all amplicons in a panel. A higher MAPD typically indicates lower coverage uniformity, which can result in missed or erroneous CNV calls. The quality control parameter metrics are summarized in S1 Table.

### BRCA1/2 pathogenic variants

The concordance rate for BRCA1/2 pathogenic variants between the SOPHiA DDM HRD Solution and the OCA Plus was 95.9%. The discordant cases included two frameshift variants at homopolymer sequences. The OCA Plus pipeline filtered out these pathogenic variants due to an unusual prediction filter that measured the amount of strand bias according to the manufacturer’s specifications. We restored these two frameshift variants by modifying the parameter of the unusual prediction filter. The other discordant variant was a long deletion, which could not be detected because it spanned the ends of amplicons. The pathogenic variants found in BRCA1/2 are listed in S2 Table.

### Selecting model and performance estimation

**Fig 1:**
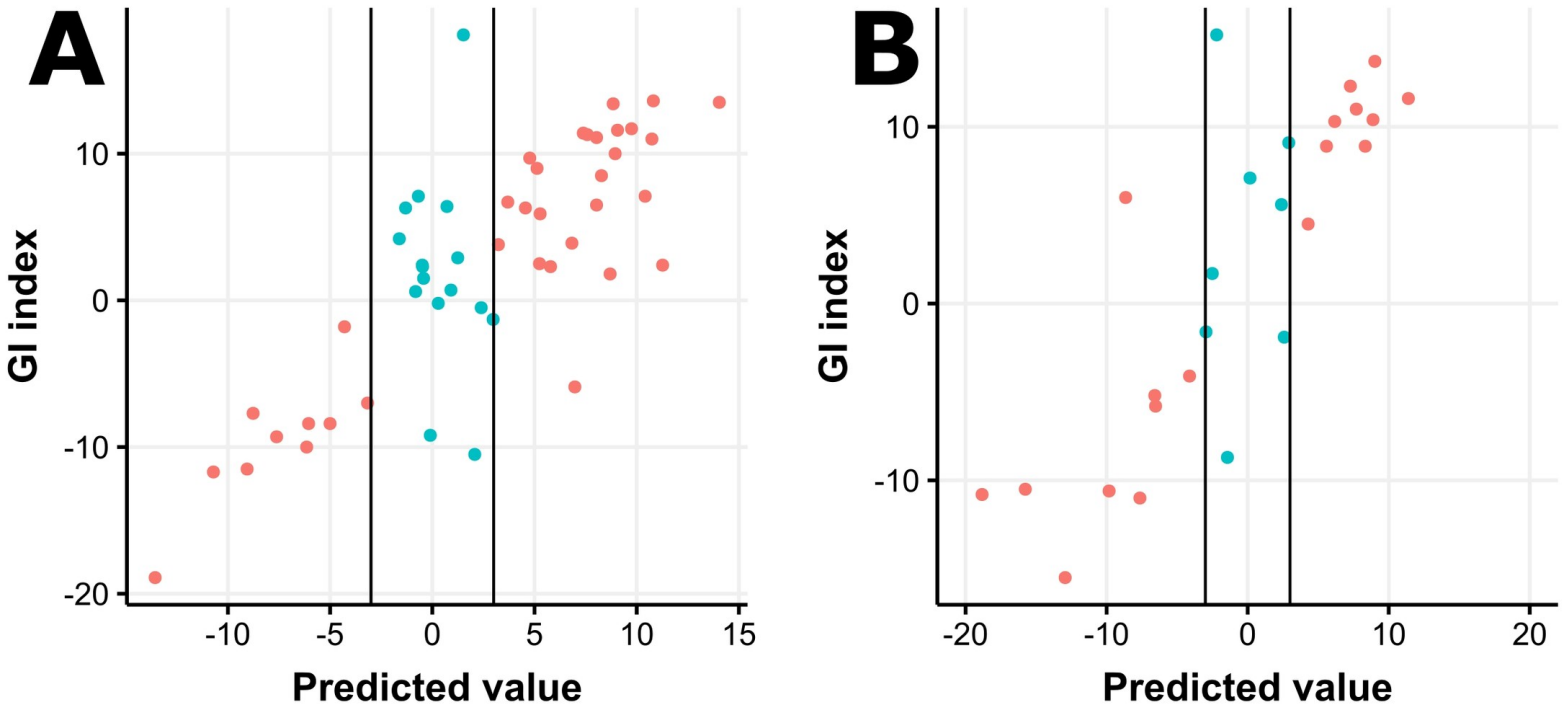
Performance of the homologous recombination deficiency (HRD) prediction model on the training set (A) and the test set (B). The black vertical lines represent borderline cut-off values (-3 and 3).

After excluding cases without a GI index from the SOPHiA DDM HRD Solution, the training set and the test set consisted of 51 and 26 cases, respectively. The model with a penalty of 0.1 and a mixture of 1 (Lasso regression) demonstrated the best performance in terms of the AUROC in a repeated fivefold cross-validation (S2 Fig). We fit the final model with the selected hyperparameters by using the entire training set. The final model equation is -21.77 + 0.200 × GIM + 0.102 × LOH(%) + 0.037 × TAI(%) + 0.261 × LST (%). To improve the performance of the prediction model, we added a borderline result category to the GI results. We classified cases with predicted values between -3 and 3 as borderline (Fig 1). The accuracy of our HRD status prediction model was 0.947 for the training set and 0.958 for the test set. Detailed performance metrics are summarized in Table 2.

**Table 2:** Performance metrics.

HRD test	N	Accuracy	Sensitivity	Specificity	Positive Predictive Value	Negative Predictive Value	F1 score
**HRD result (Train)**	38	0.947 95% CI (0.823–0.994)	0.964	0.900	0.964	0.900	0.964
**HRD result (Test)**	24	0.958 95% CI (0.789–0.999)	0.938	1.000	1.000	0.889	0.968
**HRD result (GIM)**	79	0.911 95% CI (0.826–0.964)	0.964	0.783	0.915	0.900	0.939
**GI result (Train)**	33	0.97 95% CI (0.842–0.999)	1.000	0.909	0.957	1.000	0.978
**GI result (Test)**	23	0.87 95% CI (0.664–0.972)	0.786	1.000	1.000	0.750	0.880
**GI result (GIM)**	77	0.883 95% CI (0.79–0.945)	0.960	0.741	0.873	0.909	0.914

HRD, homologous recombination deficiency; HRD result (Train/Test), prediction model performance for HRD result of SOPHiA DDM HRD Solution; GI, Genomic Integrity; GI Result (Train/Test), prediction model performance for GI result of SOPHiA DDM HRD Solution; F1 score, harmonic mean of the precision and recall; GIM, Genomic Instability Metric; HRD result/GI result (GIM), prediction performance of GIM for HRD result/GI result of SOPHiA DDM HRD Solution

### GIM

**Fig 2:**
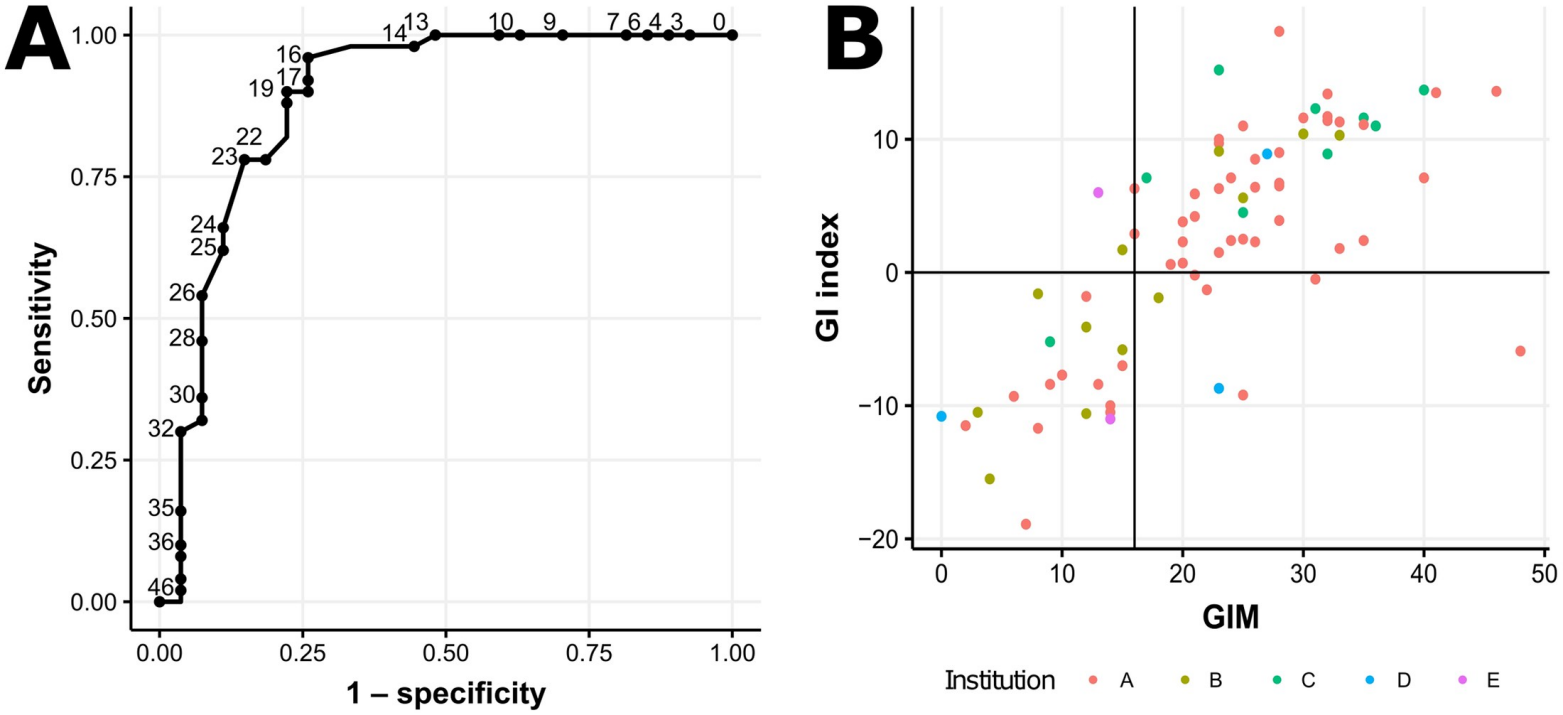
Receiver operating characteristic curve (ROC) for the genomic instability (GI) status of the SOPHiA DDM HRD Solution (A). The threshold (cut-off value) was set at 16. The black vertical line indicates a genomic instability metric (GIM) of 16. The black horizontal line indicates a GI index of 0.

The AUROC for the GIM of the OCA Plus predicting the GI status of the SOPHiA DDM HRD Solution was 0.887 (Fig 2A). We set the positive cut-off value at 16 (Fig 2B). The accuracy of the HRD status using the GIM with a cut-off value of 16 was 0.911. Detailed performance metrics are summarized in Table 2.

## Discussion

In this study, we developed a penalized linear regression model using the OCA Plus, which showed a high concordance rate with the SOPHiA DDM HRD Solution. We also observed that the GIM of the OCA Plus demonstrated high accuracy compared with the SOPHiA DDM HRD Solution. Despite being independently developed by different manufacturers, the GIM of the OCA Plus and the GI index of the SOPHiA DDM HRD Solution exhibited a high concordance rate. These findings suggest that both tests capture the same tumor characteristic, namely genomic alteration associated with HRD. When two different tests yield the same results, it reinforces the certainty of the results. It also indicates that both tests are reliable and reproducible.

It is important to note that neither the OCA Plus nor the SOPHiA DDM HRD Solution has been validated as a biomarker for PARP inhibitor response through clinical trials. Both the OCA Plus and the SOPHiA DDM HRD Solution require clinical validation through a clinical trial or a concordance test with a Food and Drug Administration–approved HRD test.

The SOPHiA DDM HRD Solution exhibited a considerable rate of failure, resulting in an undetermined result. This failure rate is similar to that of the myChoice HRD Plus assay [15]. Additionally, the OCA Plus fails to analyze HRD scores, and our prediction model relies on the OCA Plus HRD scores. Our prediction model includes a borderline category, which does not definitively determine the GI status. However, the GIM also demonstrated high accuracy without the need for a borderline category.

In three cases (4%), the OCA Plus failed to detect BRCA1/2 pathogenic variants. Two of these were c.2175del [chr13:32910667del] and c.3503dup [chr17:41244048dup] and were filtered out due to the application of a filter related to strand bias originating from homopolymer sequences. We restored these false negatives by modifying the filter parameter. The remaining one is the c.2593_2621del [chr17:41244928_41244956del] mutation, a 26 base pair deletion located within the overlapping regions of the OCA Plus amplicons. This type of long deletion seems to interfere with the generation of libraries containing both amplicons carrying this mutation, resulting in the absence of sequencing reads. The coverage depth of these two amplicons is relatively low compared with the adjacent amplicon positions. We anticipate that detecting this mutation with the OCA Plus would be challenging. Therefore, interpretation of the BRCA1/2 status results should consider the limitations of the test. These two cases had high GI and were classified as HRD positive.

Because the NGS study was not conducted on all patients with high-grade ovarian serous carcinoma, the patients included in this study may exhibit bias. However, the rates of BRCA1/2 pathogenic variant presence and positive HRD status are similar to those reported in a clinical trial of ovarian high-grade serous carcinoma using the myChoice HRD Plus assay (Myriad Genetic Laboratories).

We developed the penalized linear regression model by using a small training set and validated it with a small test set. This approach may lead to a model that is either too simplistic and underfits the data or too complex and overfits the data.

The OCA Plus offers several advantages compared with HRD-specific tests. It enables comprehensive analysis of genetic alterations, including single nucleotide variants (SNVs), insertions and deletions (indels), CNVs, structural variations, the tumor mutation burden, mismatch repair deficiency, and microsatellite instability. This broad coverage enhances the ability to identify potential targeted treatments. The high accuracy between the OCA Plus and the SOPHiA DDM HRD Solution supports its potential as a biomarker for predicting the PARP inhibitor response and its application in clinical trials for PARP inhibitors. Additionally, our study provides a cut-off value for the GIM of the OCA Plus that correlates with the SOPHiA DDM HRD Solution, with a high accuracy of 0.911.

The HRD test was developed and validated by using the genomic data from The Cancer Genome Atlas (TCGA), which includes a diverse range of ethnicities. While we examined the concordance rates between the two tests in Asian patients, we believe that our study results are applicable to a broader range of ethnicities, and not just Asian patients.

To ensure consistency among various HRD tests, it is crucial to develop a harmonized model [19]. Establishing standardized procedures would enhance reliability and comparability, promoting more robust and consistent HRD assessments across different settings and advancing precision oncology. While our research lacks specific data on suboptimal conditions, we acknowledge the importance of high DNA quality for reliable molecular testing. Caution is advised in interpreting results obtained with DNA of compromised quality.

## Conclusions

This study presents a homologous recombination deficiency prediction model from the OCA Plus that correlates with the SOPHiA DDM HRD Solution. This study provides evidence that the OCA Plus provides reliable biomarkers for homologous recombination.

## Supporting information

S1 Fig Flowchart of the study. (JPG)

S2 Fig Hyperparameter tuning and performance assessment in repeated 5-fold cross-validation on a grid of hyper-parameters. The x-axis is a penalty scaling parameter: λ (10^−5^, 10^−4^, 10^−3^, 10^−2^, and 10^−1^), color is mixture hyperparameter of penalty function: α (0.0, 0.25, 0.5, 0.75, 1.0). CCC: concordance correlation coefficient, RMSE: root mean squared error.
(JPG)

S1 Table The quality control parameter metrics of Oncomine Comprehensive Assay Plus. SD: standard deviation, MAPD: median absolute pairwise difference.
(DOCX)

S2 Table List of BRCA1/2 pathogenic variants. OCA: Oncomine Comprehensive Assay, NA: not applicable.
(DOCX)

S3 Table Data set used for analysis. (CSV)
