# Supplementary figures and images for: Prediction of homologous recombination deficiency from Oncomine Comprehensive Assay Plus correlating with SOPHiA DDM HRD Solution
Source: PLoS One. 2024 Mar 25;19(3):e0298128. doi: 10.1371/journal.pone.0298128 (PMC10962813; doi:10.1371/journal.pone.0298128)

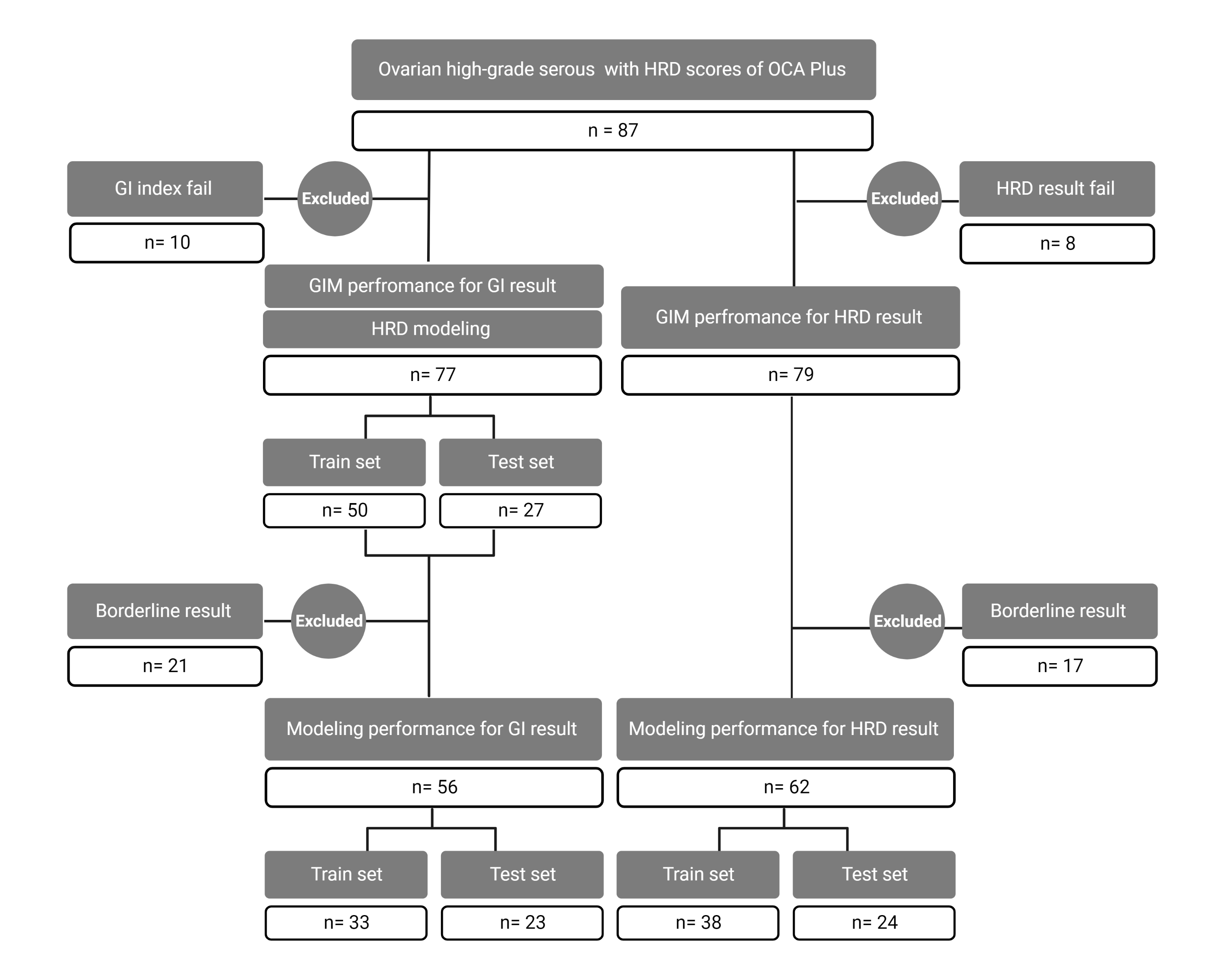

Supplement: S1 Fig — (JPG) [file pone.0298128.s001.jpg]

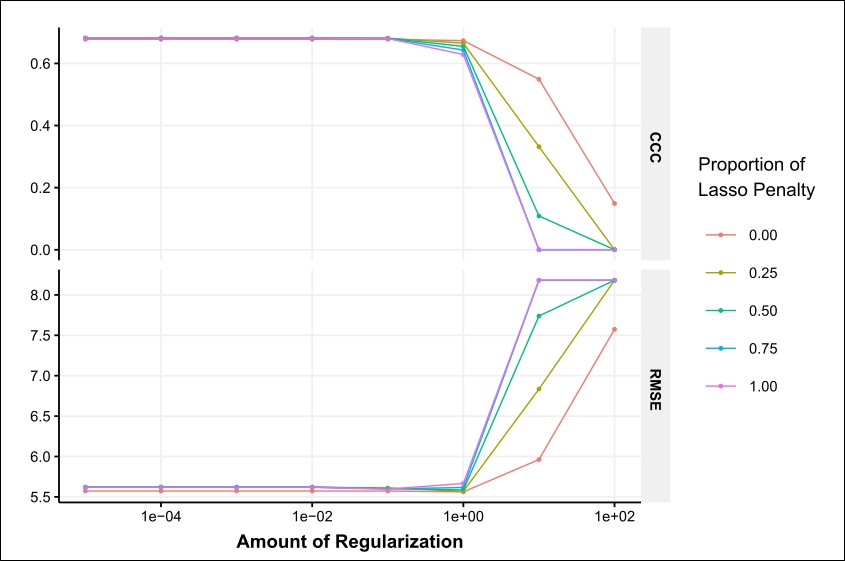

Supplement: S2 Fig — The x-axis is a penalty scaling parameter: λ (10−5, 10−4, 10−3, 10−2, and 10−1), color is mixture hyperparameter of penalty function: α (0.0, 0.25, 0.5, 0.75, 1.0). CCC: concordance correlation coefficient, RMSE: root mean squared error. (JPG) [file pone.0298128.s002.jpg]
