# Supplementary material for: Prediction of homologous recombination deficiency from Oncomine Comprehensive Assay Plus correlating with SOPHiA DDM HRD Solution
Source: PLoS One. 2024 Mar 25;19(3):e0298128. doi: 10.1371/journal.pone.0298128 (PMC10962813; doi:10.1371/journal.pone.0298128)
Supplement: S1 Table — SD: standard deviation, MAPD: median absolute pairwise difference. (DOCX) [file pone.0298128.s003.docx]

S1 Table. The quality control parameter metrics of Oncomine Comprehensive Assay Plus

|  | Overall | training | test | p value |
| --- | --- | --- | --- | --- |
| Number of cases | 87 | 55 | 32 |  |
| Tumor cellularity (%) (mean (SD)) | 67.52 (17.71) | 69.51 (15.16) | 64.34 (21.02) | 0.198 |
| Average base coverage depth (mean (SD)) | 2469.64 (462.08) | 2348.31 (481.15) | 2678.19 (343.58) | 0.001 |
| MAPD (mean (SD)) | 0.24 (0.06) | 0.24 (0.06) | 0.25 (0.06) | 0.359 |

SD: standard deviation, MAPD: median absolute pairwise difference
