# Supplementary material for: Prediction of homologous recombination deficiency from Oncomine Comprehensive Assay Plus correlating with SOPHiA DDM HRD Solution
Source: PLoS One. 2024 Mar 25;19(3):e0298128. doi: 10.1371/journal.pone.0298128 (PMC10962813; doi:10.1371/journal.pone.0298128)
Supplement: S2 Table — OCA: Oncomine Comprehensive Assay, NA: not applicable. (DOCX) [file pone.0298128.s004.docx]

S2 Table. List of BRCA1/2 pathogenic variants

| Gene | Nucleotide change (SOPHiA) | Nucleotide change (OCA Plus) | Amino acid change (SOPHiA) | Amino acid change (OCA Plus) |
| --- | --- | --- | --- | --- |
| BRCA1 | c.4186C>T | c.4186C>T | p.Gln1396* | p.Gln1396Ter |
| BRCA2 | c.7322del | c.7322del | p.Gly244Alafs*26 | p.Gly244AlafsTer26 |
| BRCA1 | c.3627dup | c.3627dup | p.Glu1210Argfs*9 | p.Glu1210ArgfsTer9 |
| BRCA1 | c.922_924delinsT | c.922_924delAGCinsT | p.Ser308* | p.S308X |
| BRCA2 | c.2175del | Not detected | p.Val726Phefs*4 | Not detected |
| BRCA1 | c.4318G>T | c.4318G>T | p.Glu1440* | p.Glu1440Ter |
| BRCA1 | c.2593_2621del | Not detected | p.Lys865Serfs*28 | Not detected |
| BRCA2 | c.5576_5579del | c.5576_5579delTTAA | p.Ile1859Lysfs*3 | p.Ile1859LysfsTer3 |
| BRCA2 | c.6239T>G | c.6239T>G | p.Leu2080* | p.Leu2080Ter |
| BRCA1 | c.3593T>A | c.3593T>A | p.Leu1198* | p.Leu1198Ter |
| BRCA1 | c.922_924delinsT | c.922_924delAGCinsT | p.Ser308* | p.Ser308Ter |
| BRCA2 | c.5576_5579del | c.5576_5579delTTAA | p.Ile1859Lysfs*3 | p.Ile1859LysfsTer3 |
| BRCA1 | c.3503dup | Not detected | p.Asn1168Lysfs*2 | Not detected |
| BRCA1 | c.4484+1G>T | c.4484+1G>T | NA | NA |
| BRCA1 | c.3442del | c.3442delG | p.Glu1148Argfs*7 | p.Glu1148ArgfsTer7 |
| BRCA2 | c.8143A>T | c.8143A>T | p.Lys2715* | p.Lys2715Ter |
| BRCA1 | c.4485-1G>T | c.4485-1G>T | NA | NA |
| BRCA1 | c.3296del | c.3296delC | p.Pro1099Leufs*10 | p.Pro1099LeufsTer10 |
| BRCA1 | c.922_924delinsT | c.922_924delAGCinsT | p.Ser308* | p.Ser308Ter |
| BRCA1 | c.3875dup | c.3875_3876insC | p.Ala1293Cysfs*2 | p.Ala1293CysfsTer2 |
| BRCA2 | c.6449_6450del | c.6449_6450delAA | p.Lys2150Serfs*25 | p.Lys2150SerfsTer25 |
| BRCA1 | c.2062A>T | c.390C>A | p.Thr688Ser | p.Tyr130Ter |
| BRCA1 | c.4629del | c.4629delG | p.Pro1544Hisfs*4 | p.Pro1544HisfsTer4 |
| BRCA1 | c.4986+5G>A | c.4986+5G>A | NA | NA |
| BRCA2 | c.7008-1G>T | c.7008-1G>T | NA | NA |
| BRCA2 | c.5576_5579del | c.5576_5579delTTAA | p.Ile1859Lysfs*3 | p.Ile1859LysfsTer3 |
| BRCA2 | c.7516C>T | c.7516C>T | p.Gln2506* | p.Gln2506Ter |
| BRCA2 | c.3599_3600del | c.3599_3600delGT | p.Cys1200* | p.Cys1200Ter |

OCA: Oncomine Comprehensive Assay
